# Association between sleep duration and chronic musculoskeletal pain in US adults: a cross-sectional study

**DOI:** 10.3389/fmed.2024.1461785

**Published:** 2024-09-25

**Authors:** Chong Li, Huaping Huang, Qingjie Xia, Li Zhang

**Affiliations:** ^1^Department of Osteoporosis, The First People’s Hospital of Kunshan Affiliated with Jiangsu University, Kunshan, China; ^2^Department of Graduate Office, The First People’s Hospital of Kunshan Affiliated with Jiangsu University, Kunshan, China; ^3^Department of Anesthesiology, The First People’s Hospital of Kunshan Affiliated with Jiangsu University, Kunshan, China

**Keywords:** sleep duration, chronic musculoskeletal pain, low back pain, NHANES, cross-sectional study

## Abstract

**Background:**

This study aims to explore the association between sleep duration and the prevalence of chronic musculoskeletal pain (CMP).

**Methods:**

A cross-sectional study was conducted using data from the National Health and Nutrition Examination Survey (NHANES) 2009–2010, which involved multiple centers across the United States. The study included 3,904 adults selected based on age and complete data availability. Demographic variables such as gender, age, race, and socioeconomic status (represented by the poverty-to-income ratio) were considered.

**Results:**

Of the participants, 1,595 reported less than 7 h of sleep, 2,046 reported 7–8 h, and 263 reported more than 9 h of sleep. Short sleep duration was associated with higher odds of CMP (OR, 1.611, 95% CI: 1.224–2.120, *p* = 0.005). Long sleep duration also showed a higher prevalence (OR, 1.751; 95% CI, 0.923 to 3.321; *p* = 0.059), although this result was not statistically significant. A U-shaped relationship emerged (Effective degree of freedom (EDF) = 3.32, *p* < 0.001), indicating that 7 h of sleep was associated with the lowest odds of CMP. In individuals with sleep durations less than 7 h, each hour increment correlated with 22.8% reduced odds of CMP (OR, 0.772; 95% CI, 0.717–0.833; *p* = 0.002). Beyond 7 h, each hour increment was associated with 38.9% increased odds of CMP (OR, 1.389; 95% CI, 1.103–1.749; *p* = 0.049).

**Conclusion:**

The findings suggest that both insufficient and excessive sleep durations are linked to a higher prevalence of CMP, highlighting the importance of optimal sleep duration for musculoskeletal health.

## 1 Introduction

Chronic musculoskeletal pain (CMP) affects over 20–33% of the global population, according to the World Health Organization (WHO), resulting in significant health and socio-economic burdens (1). Sleep plays a crucial role in maintaining physical well-being, with an optimal duration generally ranging from 7 to 9 h (2–4). Poor sleep habits are linked to various adverse health effects, including cardiovascular events (2, 5, 6), diabetes mellitus (DM) (5), Alzheimer’s Disease (4, 7), and increased mortality risk (2).

In the United States, about one-third of the population frequently experiences sleep difficulties, a condition even more prevalent among those with concurrent pain symptoms (8, 9). Observational data indicate a link between sleep disturbances and the onset, progression, and persistence of pain symptoms (8, 10–12). Chronic pain and sleep disturbances often co-occur, highlighting a clinically important reciprocal relationship. Various epidemiological studies have indicated that poor sleep quality and not getting enough sleep are correlated with a higher prevalence of chronic pain (10, 13). Conversely, experiencing chronic pain can also disrupt an individual’s sleep patterns (14).

Understanding the potential mechanisms behind this reciprocal relationship is crucial for developing new treatments for chronic pain. However, the precise relationship between sleep duration and chronic pain remains unclear. Most studies suggest a link between short sleep duration and higher prevalence of chronic pain (14), but research demonstrating a connection between excessive sleep and chronic pain is limited. This study aims to conduct a large-scale population study to explore the relationship between sleep duration and chronic pain by analyzing retrospective data from the National Health and Nutrition Examination Survey (NHANES). Specifically, we assessed the link between self-reported sleep duration and the presence of chronic pain and investigate the potential effects of age, gender, Body Mass Index (BMI), race, poverty-to-income ratio (PIR), alcohol use, smoking, DM and sleep disorders. This research offers valuable insights into understanding the intricate relationship between sleep and chronic pain, with implications for chronic pain prevention and treatment.

## 2 Methods

### 2.1 Study population

The protocols for NHANES, a cross-sectional survey, were approved by the ethics review board of the National Center for Health Statistics, and all participants provided written informed consent. It was in accordance with the policy of the National Institutes of Health that analysis conducted using de-identified data not directly involving participants was not classified as a human subject study, thus not requiring institutional review board review.

NHANES participants undergo demographic assessments, laboratory examinations, and extensive interviews, covering measures of sex, age, race, PIR, clinical profiles, and personal behaviors. The sampling structure of NHANES allows it to effectively represent the non-institutionalized population of the United States after adjustments for sex, age, race, and ethnicity were made. We primarily focused on two indicators, including sleep behavior and musculoskeletal pain. Data from NHANES 2009–2010 which included an arthritis questionnaire were obtained. We excluded 48 participants who were pregnant. Participants missing information on sleep duration, pain, or other important covariates (e.g., smoking, alcohol consumption, diabetes, PIR) were excluded. Finally, data from 3,904 participants were included in the analysis (Supplementary Figure S1).

### 2.2 Measurement

#### 2.2.1 Outcome ascertainment

CMP is defined as pain persisting or recurring for more than 3 months (15). To define this group, pain-related questions from the Arthritis Questionnaire Section (ARQ) provides interview data were utilized. Participants were classified into chronic musculoskeletal pain group if they reported experiencing “neck pain,” “upper back pain,” “mid back pain,” “low back pain,” “buttocks pain,” “hip pain,” or “rib cage pain” for at least three months.

#### 2.2.2 Exposure measurement

Participants provided self-reported information regarding their typical sleep duration on weekdays or workdays. Between 2009 and 2010, NHANES participants were requested to disclose their usual sleep duration on weekdays or workdays with the question: “How much sleep do you usually get at night on weekdays or workdays?” The distribution of the number of participants in each sleep duration group is shown in Supplementary Figure S2. The sleep duration data was segmented into three categories: less than 7 h, 7 to 8 h, and 9 h or more.

#### 2.2.3 Covariate assessment

Covariates were selected based on existing literature. Standardized questionnaires were utilized to gather data on various factors, including: age, sex, race, education level, PIR, BMI, waist circumference, smoking habits, alcohol consumption, hypertension, diabetes, sleep disorders.

Race was classified as Mexican American, other Hispanic, non-Hispanic white, non-Hispanic black, or other. Education level was categorized as below 12th grade, high school graduate or equivalent, and college or above.

Height, weight, and blood pressure were measured following established protocols. BMI was computed by dividing weight in kilograms by height in meters squared (kg/m^2).

Participants were grouped as never smokers, former smokers or current smokers based on their responses regarding smoking history and current behavior. Alcohol consumption status was determined by the question “Have you had at least 12 drinks of any type of alcoholic beverage in any one year?.” Hypertension was identified by self-reporting a history of high blood pressure diagnosis. Diabetes was established through self-reporting a diagnosis of diabetes or sugar diabetes, excluding gestational diabetes. Sleep Disorders was defined based on the questions “Have you ever told a doctor you had trouble sleeping?” and “Have you ever been told by a doctor you have a sleep disorder?.” These covariates were included to account for potential confounding variables in the relationship between sleep duration and pain.

### 2.3 Statistical analysis

This study utilized sample weights provided by NHANES for weighted analysis to accurately reflect the actual distribution of the U.S. population, ensuring a high level of accuracy and representativeness in assessing the connection between sleep duration and chronic pain. The data was presented as survey-weighted mean (95% confidence interval [CI]) for continuous variables and survey-weighted percentage (95% CI) for categorical variables. The population with missing data in the variables was excluded from the analysis. A comparison of baseline characteristics was conducted among participants with different sleep durations (< 7 h, 7 to 8 h, and ≥ 9 h) using one-way ANOVA for continuous variables and the chi-square test for categorical variables.

We selected these confounders on the basis of their association with the outcomes of interest or changes in effect estimates of more than 10% (16). After considering the clinical significance, we adjusted for the following covariates: age (years), sex, BMI, race, PIR, alcohol consumption, smoking habits, diabetes, and sleep disorders. Odds ratios (ORs) and 95% confidence intervals (CIs) were computed for prevalent CMP and low back pain (LBP) using weighted logistic regression model. To further assess the impact of excluding missing data, we conducted a sensitivity analysis. We compared the results from the complete dataset with those from the dataset after excluding individuals with missing data. To assess the nonlinear relationship between sleep duration and prevalent CMP and LBP, a generalized additive model (GAM) was employed. The effective degrees of freedom (EDF) provide insight into the curvature of the smoothing curve (17, 18). An EDF value of 1 indicates a linear relationship, while an EDF value greater than 1 suggests a more complex relationship between chronic pain and sleep duration. By analyzing the smoothing curve, a weighted two-piecewise linear regression model (19, 20) was developed to identify any potential threshold effect. The model adjusted for possible confounders, and the results were presented as odds ratios (ORs) with corresponding 95% CIs. Additionally, we conducted stratified analyses for gender, age, smoking, BMI, diabetes, and sleep disorders to validate the results.

All analyses were conducted using the R statistical software (The R Foundation^1^) and EmpowerStats software (X&Y solutions, Inc. Boston MA^2^) running on R software. A two-sided *p*-value < 0.05 was considered statistically significant.

## 3 Results

### 3.1 Baseline characteristics of the study participants

Among the 3,904 participants in the study, 1,956 were male and 1,948 were female. Sleep duration is categorized into three distinct groups: those sleeping less than 7 h, those sleeping 7 to 8 h, and those sleeping 9 h or more. Compared with the group that slept for 7 to 8 h, participants with shorter sleep durations were more often male, had a lower PIR, and exhibited higher BMI or waist circumferences. They were also more likely to smoke and to have comorbid hypertension, diabetes, and sleep disturbances. In contrast, participants with longer sleep durations, as opposed to the 7–8 h group, were predominantly female, had a lower PIR, and had lower BMI or waist circumferences. They were also smokers and frequently had diabetes, but less frequently had hypertension and reported alcohol use. A detailed summary of the baseline characteristics of the subjects is provided in Table 1.

**Table 1:** Selected characteristics of NHANES 2009–2010 participants 20 ≤ aged ≤69 years (*n* = 3,904).

Characteristics	All participants (*n* = 3,904)	Short sleep (< 7 h) (*n* = 1,595)	Normal sleep (7–8 h) (*n* = 2046)	Long sleep (≥ 9 h) (*n* = 263)	*P*-value
Gender (%)					0.0204
Male	50.57 (49.01, 52.13)	53.66 (49.87, 57.40)	49.32 (46.87, 51.78)	43.70 (37.80, 49.79)	
Female	49.43 (47.87, 50.99)	46.34 (42.60, 50.13)	50.68 (48.22, 53.13)	56.30 (50.21, 62.20)	
Age (year)	43.61 (42.87, 44.35)	43.95 (43.00, 44.89)	43.47 (42.43, 44.50)	42.93 (40.87, 45.00)	0.6654
Age, *n* (%)					0.2926
< 40	39.84 (37.15, 42.60)	38.33 (34.77, 42.01)	40.87 (37.18, 44.66)	39.45 (32.36, 47.02)	
40–65	53.15 (50.63, 55.66)	55.44 (52.25, 58.59)	51.89 (48.59, 55.18)	51.14 (44.58, 57.65)	
≥65	7.00 (6.17, 7.94)	6.23 (4.79, 8.08)	7.24 (5.95, 8.77)	9.41 (5.88, 14.73)	
BMI (kg/m^2^)	28.92 (28.62, 29.22)	29.51 (28.99, 30.04)	28.61 (28.16, 29.07)	28.20 (27.17, 29.22)	0.0301
BMI, *n* (%)					0.0043
<18.5	1.80 (1.22, 2.65)	2.21 (1.58, 3.09)	1.50 (0.95, 2.36)	2.19 (0.70, 6.64)	
18.5–25	28.37 (25.26, 31.71)	24.44 (21.29, 27.89)	30.09 (25.60, 35.01)	35.92 (28.09, 44.58)	
25–30	33.08 (30.60, 35.65)	31.73 (29.08, 34.50)	34.37 (29.44, 39.65)	29.22 (21.92, 37.78)	
>30	36.75 (34.58, 38.97)	41.62 (37.96, 45.38)	34.04 (30.47, 37.80)	32.67 (25.00, 41.40)	
Waist	98.32 (97.43, 99.22)	99.87 (98.43, 101.31)	97.53 (96.37, 98.69)	96.40 (93.92, 98.88)	0.0170
Race/ethnicity, *n* (%)					<0.0001
Mexican American	8.39 (4.71, 14.48)	8.28 (4.85, 13.78)	8.29 (4.60, 14.47)	9.90 (4.21, 21.57)	
Other Hispanic	4.75 (2.81, 7.91)	5.79 (3.68, 8.99)	4.00 (2.20, 7.17)	5.40 (2.46, 11.47)	
Non-Hispanic White	69.96 (62.54, 76.46)	63.05 (55.32, 70.16)	74.51 (67.07, 80.75)	69.26 (56.99, 79.30)	
Non-Hispanic Black	10.79 (9.14, 12.70)	16.07 (13.64, 18.84)	7.38 (5.95, 9.13)	10.74 (6.71, 16.76)	
Other Race	6.12 (4.62, 8.05)	6.82 (4.92, 9.37)	5.82 (4.24, 7.94)	4.69 (2.39, 9.01)	
Education, *n* (%)					<0.0001
Less than 12th grade	16.10 (14.40, 17.96)	17.51 (16.00, 19.14)	14.69 (12.63, 17.03)	20.56 (14.80, 27.82)	
High school graduate or equivalent	22.70 (20.35, 25.24)	26.33 (22.86, 30.12)	19.86 (17.27, 22.74)	27.25 (20.66, 35.00)	
Some college or above	61.20 (58.05, 64.26)	56.16 (52.97, 59.29)	65.44 (61.30, 69.36)	52.20 (43.40, 60.86)	
PIR	3.09 (3.00, 3.18)	2.92 (2.80, 3.04)	3.23 (3.12, 3.33)	2.88 (2.62, 3.13)	<0.0001
Alcohol status (Yes), *n* (%)	80.51 (78.06, 82.74)	80.02 (78.00, 81.90)	81.33 (78.27, 84.04)	75.93 (69.90, 81.08)	0.0311
Hypertension, *n* (%)	26.07 (23.89, 28.38)	29.84 (26.43, 33.48)	24.36 (20.86, 28.23)	19.45 (14.91, 24.96)	0.0084
Diabetes, *n* (%)					0.1390
Yes	7.20 (6.13, 8.45)	8.80 (6.72, 11.44)	6.23 (5.24, 7.39)	6.68 (4.21, 10.46)	
No	91.01 (89.83, 92.07)	89.35 (86.86, 91.41)	92.06 (90.76, 93.20)	91.24 (87.18, 94.10)	
Borderline	1.79 (1.36, 2.35)	1.85 (1.23, 2.78)	1.71 (0.97, 3.00)	2.08 (1.12, 3.84)	
Sleep disorders, *n* (%)	25.89 (23.02, 28.98)	35.75 (32.01, 39.67)	20.01 (17.17, 23.20)	21.35 (12.99, 33.03)	<0.0001
Smoking status (self-reported), *n* (%)					<0.0001
Never smoking	55.74 (51.78, 59.63)	50.53 (46.63, 54.42)	59.47 (54.43, 64.32)	52.44 (41.46, 63.20)	
Former smoker	22.46 (19.34, 25.92)	21.84 (19.24, 24.70)	23.47 (19.27, 28.26)	16.81 (12.23, 22.66)	
Current smoking	21.80 (20.09, 23.61)	27.63 (24.95, 30.47)	17.06 (14.96, 19.38)	30.75 (21.22, 42.25)	

PIR, poverty-to-income ratio; BMI, body mass index. For continuous variables: survey-weighted mean (95% CI), *P*-value was by survey-weighted linear regression (svyglm); For categorical variables: survey-weighted percentage (95% CI), *P*-value was by survey-weighted Chi-square test (svytable).

### 3.2 Impact of short and long sleep durations on chronic musculoskeletal pain and low back pain

Table 2 depicts the prevalence of pain in different anatomical regions. Both longer sleep durations (≥9 h) and shorter sleep durations (<7 h), compared to the standard sleep duration (7–8 h), are linked to increased pain prevalence across all examined anatomical regions (neck, upper back, mid back, low back, buttocks or hips). Table 3 shows the results of weighted logistic regression model construction between sleep duration and CMP and LBP, detailing odds ratios (ORs) and 95% CIs for both conditions across short and long sleep durations versus the normal sleep duration. In the unadjusted model, short sleep duration was associated with 97% higher odds of CMP (OR = 1.970 [95% CI 1.648, 2.354], *p* < 0.001), and long sleep duration with 79% higher odds compared to normal sleep duration (OR = 1.797 [95% CI 1.201, 2.690], *p* = 0.013). For LBP, the ORs were 1.844 ([95% CI 1.527, 2.228], *p* < 0.001) for short sleepers and 1.911 ([95% CI 1.233, 2.963], *p* = 0.012) for long sleepers. After adjusting for confounders, the adjusted ORs for CMP were 1.611 (95% CI 1.224, 2.120, *p* = 0.005) for short and 1.751 (95% CI 0.923, 3.321, *p* = 0.059) for long sleep durations. For LBP, the adjusted ORs were 1.428 (95% CI 1.085, 1.879, *p* = 0.015) for short and 1.818 (95% CI 0.883, 3.741, *p* = 0.069) for long sleep durations. Results derived from the analysis of data with missing covariates corroborated the findings observed in the full dataset of the study (Supplementary Table S1).

**Table 2:** Pain prevalence in different body regions among participants with different sleep durations.

Outcomes	All participants (*n* = 3,904)	Short sleep (< 7 h) (*n* = 1,595)	Normal sleep (7–8 h) (*n* = 2046)	Long sleep (≥ 9 h) (*n* = 263)	*P*-value
Neck pain that had lasted 3 or more months, *n* (%)	7.61 (6.64, 8.71)	10.17 (8.08, 12.73)	5.84 (4.79, 7.09)	8.70 (4.49, 16.17)	0.0020
Upper back pain that had lasted 3 or more months, *n* (%)	4.38 (3.73, 5.15)	6.33 (5.05, 7.90)	2.92 (2.01, 4.23)	6.32 (3.10, 12.43)	0.0011
Mid back pain that had lasted 3 or more months, *n* (%)	4.24 (3.54, 5.06)	6.32 (4.88, 8.14)	2.89 (2.06, 4.05)	4.20 (1.61, 10.53)	0.0018
Low back pain that had lasted 3 or more months, *n* (%)	14.23 (12.92, 15.64)	18.50 (16.06, 21.22)	10.94 (10.09, 11.86)	18.96 (12.52, 27.67)	<0.0001
Buttocks pain that had lasted 3 or more months, *n* (%)	6.71 (5.60, 8.01)	9.21 (6.81, 12.35)	5.22 (4.25, 6.41)	5.50 (3.14, 9.47)	0.0005
Hip pain that had lasted 3 or more months, *n* (%)	3.37 (2.73, 4.15)	4.54 (3.49, 5.87)	2.53 (1.77, 3.62)	4.10 (2.30, 7.20)	0.0048
Rib cage pain that had lasted 3 or more months, *n* (%)	0.83 (0.55, 1.26)	0.98 (0.58, 1.65)	0.69 (0.31, 1.55)	1.28 (0.42, 3.89)	0.5859
Chronic musculoskeletal pain, *n* (%)	21.57 (19.41, 23.90)	28.31 (24.99, 31.88)	16.70 (15.00, 18.55)	26.38 (18.08, 36.78)	<0.0001

For categorical variables: survey-weighted percentage (95% CI), *P*-value was by survey-weighted Chi-square test (svytable).

**Table 3:** Results of multiple logistic regressions on the association between sleep duration and pain outcomes.

Outcomes	Non-adjusted Model		Model 1		Model 2		Model 3	
	OR (95% CI)	*P*-value	OR (95% CI)	*P*-value	OR (95% CI)	*P*-value	OR (95% CI)	*P*-value
Chronic musculoskeletal pain								
Sleep duration	0.806 (0.758, 0.858)	<0.001	0.809 (0.759, 0.863)	<0.001	0.884 (0.829, 0.942)	0.009	0.883 (0.829, 0.942)	0.009
Hours of Sleep								
Short sleep (<7 h)	1.970 (1.648, 2.354)	<0.001	1.984 (1.651, 2.383)	<0.001	1.624 (1.349, 1.955)	0.002	1.611 (1.224, 2.120)	0.005
Normal sleep (7-8 h)	Ref		Ref		Ref		Ref	
Long sleep (≥9 h)	1.797 (1.201, 2.690)	0.013	1.822 (1.186, 2.801)	0.0181	1.774 (1.140, 2.761)	0.044	1.751 (0.923, 3.321)	0.059
Low back pain								
Sleep duration	0.819 (0.755, 0.888)	<0.001	0.823 (0.758, 0.893)	<0.001	0.913 (0.825, 1.010)	0.059	0.911 (0.823, 1.008)	0.057
Hours of Sleep								
Short sleep (<7 h)	1.844 (1.527, 2.228)	<0.001	1.843 (1.527, 2.224)	<0.001	1.447 (1.136, 1.843)	0.008	1.428 (1.085, 1.879)	0.015
Normal sleep (7-8 h)	Ref		Ref		Ref		Ref	
Long sleep (≥9 h)	1.911 (1.233, 2.963)	0.012	1.930 (1.219, 3.056)	0.0160	1.853 (0.972, 3.532)	0.049	1.818 (0.883, 3.741)	0.069

Model I: Adjust for sex, age, race. Model II: Adjust for the variables in Model I plus BMI, alcohol status, smoking status, diabetes and sleep disorders. Model III: Adjust for the variables in Model II plus poverty-to-income ratio.

### 3.3 Threshold effect analysis for sleep duration on chronic musculoskeletal pain and low back pain

**Figure 1:**
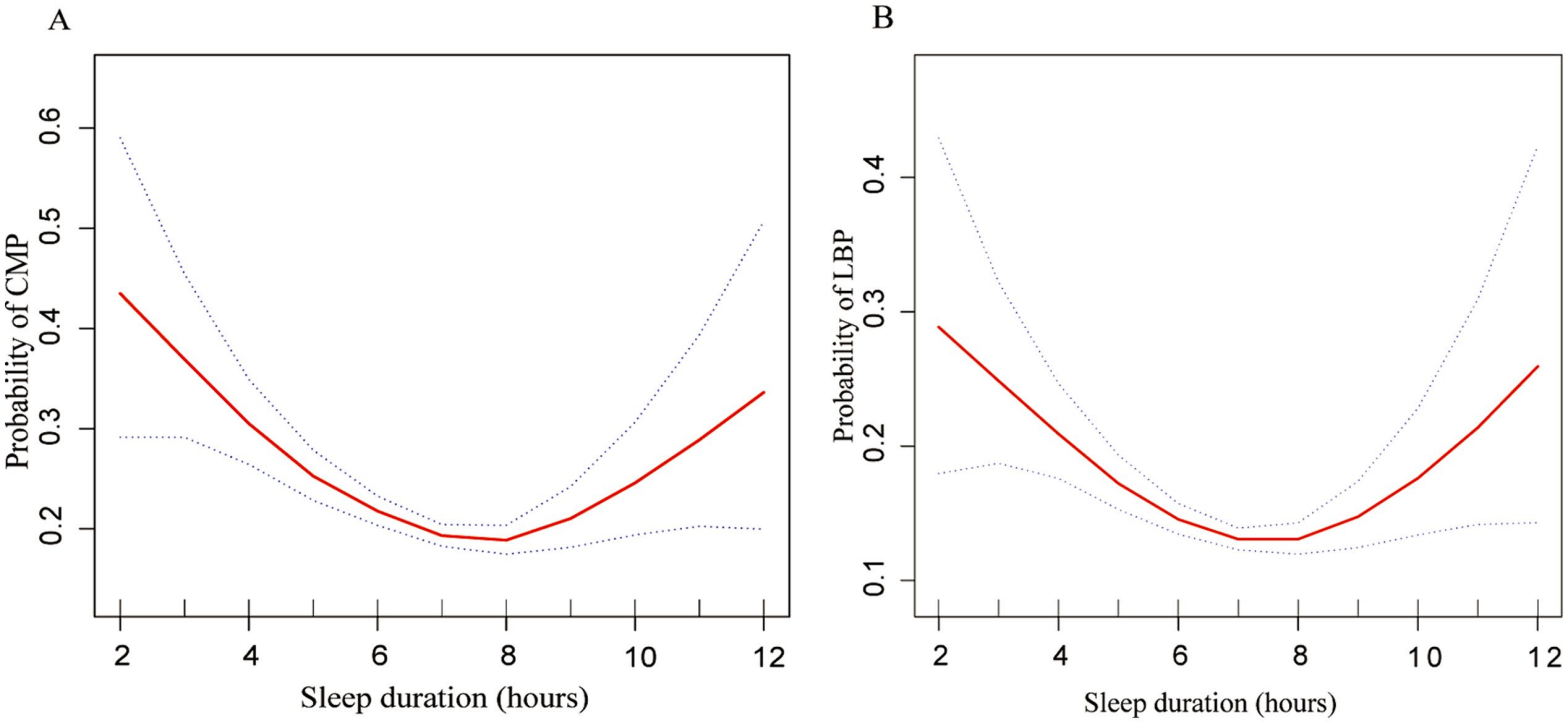
Multivariate-adjusted spline curves for relationship of sleep duration with chronic musculoskeletal pain and low back pain. **(A)** Probability of CMP and **(B)** probability of LBP. The solid line represents the estimated probability of CMP and LBP, and the dotted lines show point-wise 95% confidence intervals. These estimates are adjusted for sex, age, race, PIR, BMI, alcohol consumption, smoking habits, diabetes and sleep disorders. CMP, chronic musculoskeletal pain; LBP, low back pain; PIR, Poverty-to-Income Ratio.

Figure 1 illustrates a U-shaped relationship between CMP (EDF = 3.32, *p* < 0.001), LBP (EDF = 2.86, *p* < 0.001), and sleep duration post adjustment for confounders based on the generalized additive models. We further conducted a threshold effect analysis using the cut-off value of 7 h and found that the odds of CMP decreased with sleep duration until it reached a minimum at 7 h (OR = 0.772 [95% CI 0.717, 0.833], *p* = 0.002). Conversely, exceeding 7 h of sleep led to a significant increase in CMP odds (OR = 1.389 [95% CI 1.103, 1.749], *p* = 0.049) (Table 4). Similarly, the odds of LBP decreased with sleep duration until it reached a minimum at 7 h (OR = 0.776 [95% CI 0.663, 0.906], *p* = 0.006). However, surpassing 7 h of sleep resulted in an odds elevation (OR = 1.419 [95% CI 0.904, 2.228], *p* = 0.069).

**Table 4:** Threshold effect analysis of sleep duration on chronic musculoskeletal pain and low back pain.

Outcomes	Non-adjusted Model		Model 1		Model 2	
	OR (95% CI)	*P*-value	OR (95% CI)	*P*-value	OR (95% CI)	*P*-value
Chronic musculoskeletal pain (Yes/No)						
Sleep (≤7 h)	0.671 (0.621, 0.725)	<0.001	0.766 (0.716, 0.819)	<0.001	0.772 (0.717, 0.833)	0.002
Sleep (>7 h)	1.431 (1.153, 1.777)	0.006	1.395 (1.113, 1.750)	0.034	1.389 (1.103, 1.749)	0.049
Low back pain (Yes/No)						
Sleep (≤7 h)	0.657 (0.597, 0.722)	<0.001	0.766 (0.673, 0.872)	0.002	0.776 (0.663, 0.906)	0.006
Sleep (>7 h)	1.470 (1.157, 1.869)	0.007	1.430 (0.973, 2.102)	0.049	1.419 (0.904, 2.228)	0.069

Model I: Adjust for sex, age, race. Model II: Adjusted for sex, age, race, poverty-to-Income Ratio, BMI, alcohol status, smoking status, diabetes and sleep disorder.

**Figure 2:**
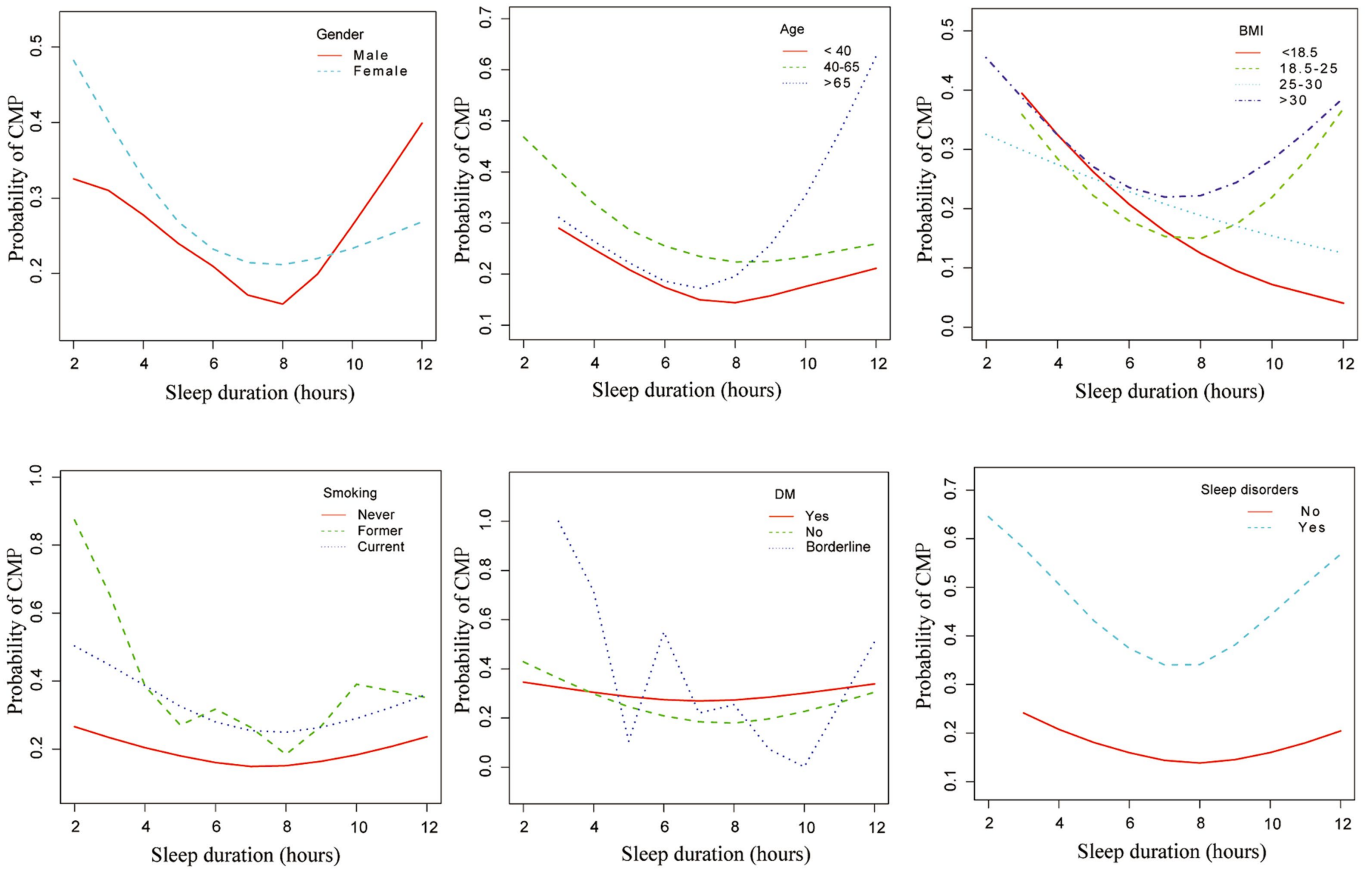
Associations between sleep duration and chronic musculoskeletal pain across different subgroups. This figure illustrates the associations between sleep duration and chronic musculoskeletal pain, stratified by gender, age, BMI, smoking status, diabetes, and sleep disorders. Adjusted for age (years), sex, race, PIR, BMI, alcohol consumption, smoking habits, diabetes and sleep disorders. CMP, chronic musculoskeletal pain; PIR, Poverty-to-Income Ratio.

When stratified by gender, age, BMI, smoking, diabetes, and sleep disorders, a curvilinear relationship between sleep duration and musculoskeletal pain was still observed. Female, aged 40–65, smokers, obese individuals, those with diabetes, and individuals with sleep disorders have a high prevalence of CMP (Figure 2).

## 4 Discussion

In this population-based cross-sectional investigation, we discovered an independent relationship between sleep duration and a higher prevalence of chronic musculoskeletal pain. Our analysis revealed a non-linear correlation between sleep duration and pain prevalence, with significant increases in pain odds observed at extreme sleep durations. The optimal sleep duration for mitigating pain odds was identified as 7 h, highlighting the importance of maintaining this balance to reduce the likelihood of chronic musculoskeletal pain.

Prior research has primarily focused on the adverse effects of insufficient sleep, with less attention given to the dangers linked with excessive sleep. Nevertheless, recent studies, including this one, have identified a U-shaped association between sleep duration and various health outcomes, including chest pain (21) and chronic musculoskeletal pain (22). This study’s results align with prior research, highlighting that both insufficient and excessive sleep are associated with higher odds of pain.

In our study, we observed a U-shaped relationship between sleep duration and the prevalence of CMP, with 7 h of sleep being associated with the lowest odds. This finding is consistent with other research demonstrating that deviations from the optimal sleep duration of 7 h are linked to various adverse health outcomes. For example, studies have shown that both insufficient and excessive sleep are associated with increased all-cause mortality (2) and accelerated phenotypic aging (23). These findings suggest that maintaining an optimal sleep duration is crucial not only for reducing the prevalence of chronic pain but also for overall health and longevity.

It is reported that the relationship between pain and sleep is complex and bidirectional: pain can disrupt sleep, while insufficient or interrupted sleep can lower pain thresholds and increase spontaneous pain levels (14). The underlying neurochemical mechanisms involved in this two-way relationship may encompass various components of the opioid, monoaminergic, orexinergic, immune, melatonin, and endocannabinoid systems. Additionally, the hypothalamic–pituitary–adrenal axis, adenosinergic signaling, and nitric oxide pathways are also thought to be implicated (14). Musculoskeletal pain is more prevalent and prone to chronicity compared to headaches or abdominal pain. Previous research has indicated that short-term sleep deprivation disrupts the internal redox balance of the body (24). Furthermore, sleep is thought to not only conserve energy but also facilitate the removal of toxic proteins and metabolic byproducts through different pathways (25). Sleep deprivation leads to oxidative stress in glial mitochondria and lipid droplet accumulation, necessitating overnight sleep for the induction of glial cell and neuronal mitochondrial autophagy. Sleep disruption has been associated with a reduction in N3 sleep while studies have indicated that partial night sleep deprivation can trigger inflammation in the body (26). The responsible mechanisms of the association among excessive sleep and musculoskeletal pain remain to be explored. But the present study demonstrates that individuals sleeping less than 7 h and 9 h or more have elevated levels of CRP and neutrophil count, indicating increased inflammation (Unpublished data). Previous research has demonstrated that CMP can lead to cognitive impairment and accelerated aging in the hippocampal region (27), supporting the rationale for investigating the association between sleep duration and CMP prevalence.

The study identified findings of borderline significance regarding the association between long sleep duration and musculoskeletal pain. Although the *p*-value of 0.059 is close to the conventional threshold for statistical significance, it suggests that further research is needed to substantiate this finding. Given the potential implications for public health and clinical practice, we recommend that future studies should consider larger sample sizes and longer follow-up periods.

Our research uncovered a more pronounced influence of sleep disturbances on pain perception in women compared to men. The underlying mechanisms behind how sleep affects pain perception, particularly in females, remain unclear. One hypothesis is that there may be biological distinctions in the way pain is regulated, driven by factors such as hormones and genetics, leading to divergent functional neurological changes in females compared to males when experiencing sleep issues (28).

The present study found a higher prevalence of musculoskeletal pain in populations with cigarette smoking. Smoking and pain have a complex relationship. Research indicates that smoking can worsen chronic pain conditions. Smokers with chronic pain tend to report higher pain intensity, functional impairment, and psychological symptoms compared to non-smokers (29). The prevalence of pain was also higher among former smokers compared to never smokers. While nicotine can initially have analgesic effects by stimulating the body’s natural pain relief system, chronic exposure to nicotine and tobacco smoke can lead to increased pain sensitivity and the need for more analgesic medication (30, 31). Smoking can impair the body’s ability to heal, decrease oxygen delivery, and lead to degenerative diseases that cause chronic pain (32). Additionally, smoking is associated with a higher odds of developing conditions like rheumatoid arthritis, which causes chronic joint pain and stiffness (32).

The present study found a higher prevalence of pain among adults aged 40–65 years compared to those under 40 years and over 65 years of age, excluding prolonged sleep durations. Several factors may contribute to the peak in pain prevalence within this middle-aged group: Many chronic diseases like osteoarthritis and degenerative spinal disease, which can lead to chronic pain, have a higher prevalence during the ages of 40–65 years (33). Adults in this age range often have high occupational stress and decreasing physical activity, predisposing them to musculoskeletal disorders (34). The perimenopausal period in women also falls within this age bracket, and hormone fluctuations may lower pain thresholds (35). Although older adults over 65 years have a high frequency of musculoskeletal conditions, pain thresholds gradually increase and pain perception declines in this elderly population (36).

## 5 Limitation

Our study boasts certain strengths, such as a relatively large sample size and adjustments for factors closely associated with musculoskeletal pain, such as smoking and alcohol consumption. However, our research also carries limitations. Firstly, the cross-sectional design of our study precludes causal inferences; thus, the directionality between sleep duration and pain requires further investigation through longitudinal or interventional research. Additionally, baseline self-reported sleep quality may be inaccurate, as individuals who self-report sleep disturbances are more likely to inaccurately estimate their sleep onset latency and total sleep time. In the future, both self-reported data and objective measurement methods such as actigraphs or sleep diaries, should be employed concurrently to enhance the accuracy of the data.

Additionally, our study solely focused on sleep duration, overlooking disruptions in sleep quality and patterns, such as fragmentation or shallow sleep, which are closely associated with heightened pain perception. Future research should incorporate monitoring and analyzing sleep patterns to comprehensively understand the relationship between sleep and pain. This study excluded participants with missing covariate data, which may result in a sample that does not fully represent the broader population. Consequently, despite our efforts to control for confounding factors in the analysis, selection bias may still influence the interpretation of our results. The study results are only applicable to the population aged 20–69 with available pain data, indicating that our findings are limited to this specific demographic in the United States.

## 6 Conclusion

To summarize, the duration of sleep was found to be linked to a higher occurrence of CMP in this study, which utilized data from the NHANES conducted between 2009 and 2010. It was observed that not only insufficient sleep, but also excess sleep, can contribute to a higher prevalence of experiencing pain.

## Data Availability

The raw data supporting the conclusions of this article will be made available by the authors, without undue reservation.

## Footnotes

^1^http://www.R-project.org

^2^www.empowerstats.com
