# Supplementary material for: Association between sleep duration and chronic musculoskeletal pain in US adults: a cross-sectional study
Source: Front Med (Lausanne). 2024 Sep 25;11:1461785. doi: 10.3389/fmed.2024.1461785 (PMC11461308; doi:10.3389/fmed.2024.1461785)

## **Association between sleep duration and chronic musculoskeletal pain in US adults: A cross-sectional study**

Supplementary files

**Supplementary Table 1.** Results of multiple logistic regressions on the association between sleep duration and pain outcomes in missing data.

**Supplementary figure 1.** Participants inclusion flowchart.

**Supplementary figure 2.** The distribution of the number of participants in each sleep duration group.

**Supplementary Table 1.** Results of multiple logistic regressions on the association between sleep duration and pain outcomes in missing data.

| **Outcomes** | OR (95% CI) | *P*-value |
| --- | --- | --- |
| **chronic musculoskeletal pain** |  |  |
| Hours of Sleep |  |  |
| Short sleep (<7h) | 2.31 (1.63, 3.26) | <0.0001 |
| Normal sleep (7-8h) | Ref |  |
| Long sleep (≥9h) | 2.03 (1.06, 3.88) | 0.0326 |
| **Low back pain** |  |  |
| Hours of Sleep |  |  |
| Short sleep (<7h) | 3.15 (2.01, 4.92) | <0.0001 |
| Normal sleep (7-8h) | Ref |  |
| Long sleep (≥9h) | 2.61 (1.18, 5.77) | 0.0181 |

Adjust model adjust for: Age, gender.

**Supplementary figure 1.** Participants inclusion flowchart.

**
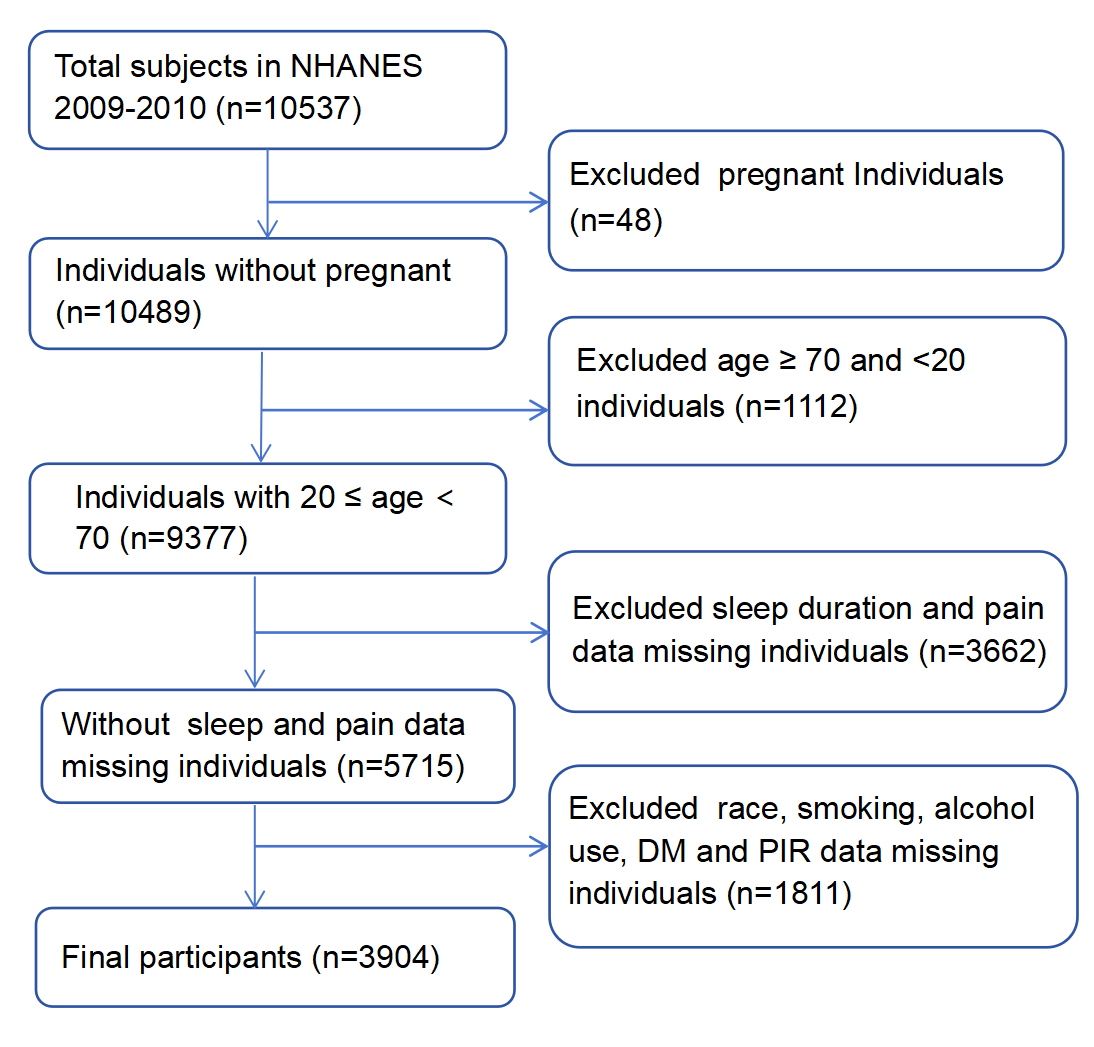
**

**Supplementary figure 2.**The distribution of the number of participants in each sleep duration group.


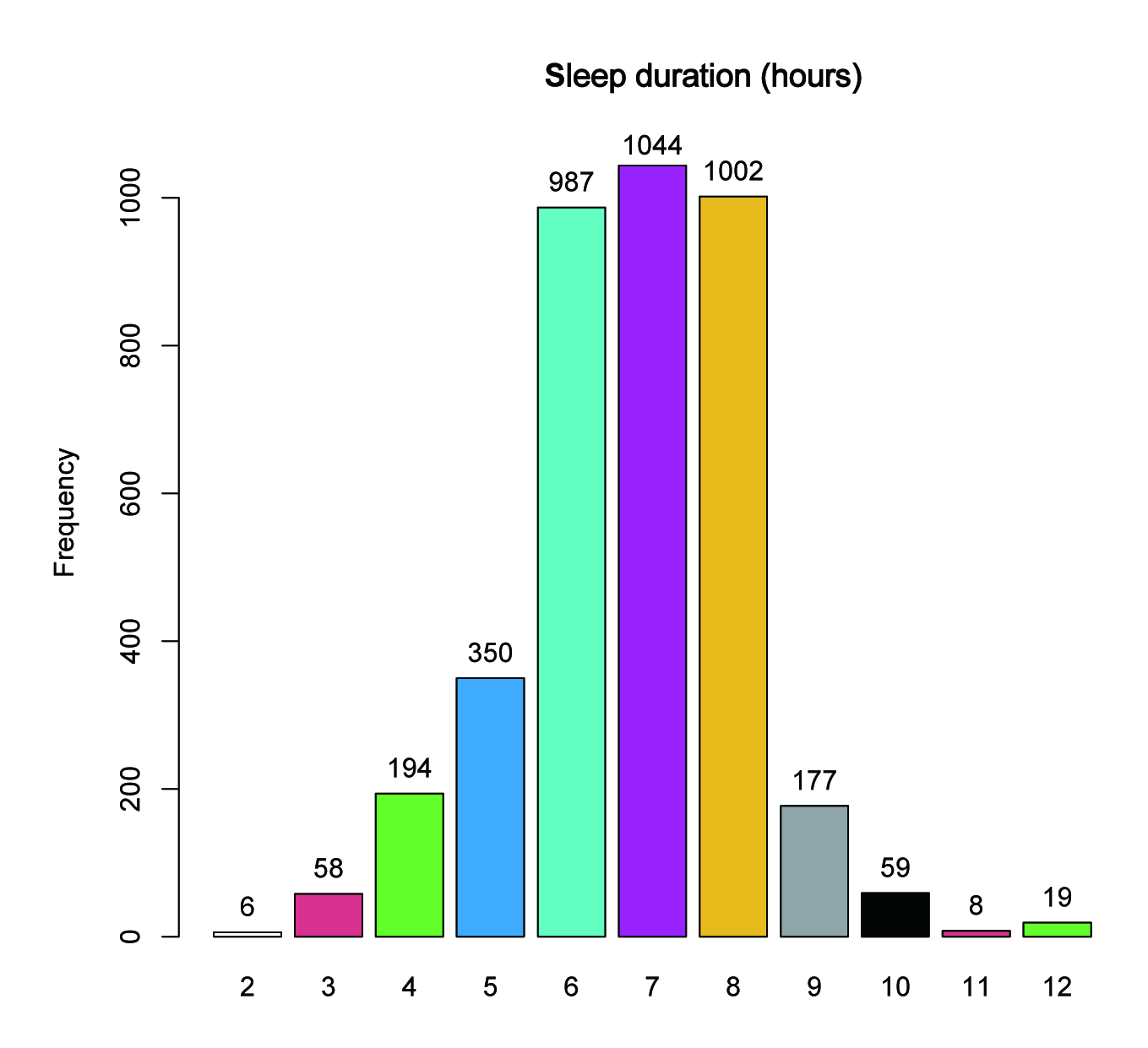

Supplement: Supplementary file 1 [file Data_Sheet_1.doc]
